# Inhibition of mTOR in bovine monocyte derived macrophages and dendritic cells provides a potential mechanism for postpartum immune dysfunction in dairy cows

**DOI:** 10.1038/s41598-022-19295-1

**Published:** 2022-09-05

**Authors:** Anja S. Sipka, Tawny L. Chandler, Thomas Weichhart, Hans-Joachim Schuberth, Sabine Mann

**Affiliations:** 1 Department of Population Medicine and Diagnostic Sciences, College of Veterinary Medicine, Cornell University, 231 Farrier Road, Ithaca, NY 14853 USA; 2 Center for Pathobiochemistry and Genetics, Medical University of Vienna, Währinger Straße 10, 1090 Vienna, Austria; 3 Institute for Immunology, University of Veterinary Medicine, Buenteweg 2, 30559 Hannover, Germany

**Keywords:** Inflammation, Innate immune cells

## Abstract

Dairy cattle experience a profound nutrient deficit postpartum that is associated with immune dysfunction characterized by heightened inflammation and reduced pathogen clearance. The activation of the central nutrient-sensing mTOR pathway is comparatively reduced in leukocytes of early postpartum dairy cows during this time of most pronounced nutrient deficit. We assessed the effect of pharmacological mTOR inhibition (Torin-1, rapamycin) on differentiation of monocyte derived classically (M1) and alternatively (M2) activated macrophages (MPh) and dendritic cells (moDC) from 12 adult dairy cows. Treatment with mTOR inhibitors generated M1 MPh with increased oxidative burst and expression of IL12 subunits but decreased phagocytosis and expression of *IL1B, IL6,* and IL10. In M2 MPh, treatment inhibited expression of regulatory features (CD163, *ARG2,* IL10) skewing the cells toward an M1-like phenotype. In moDC, mTOR inhibition increased expression of pro-inflammatory cytokines (*IL12A, IL12B, IL1B, IL6*) and surface CD80. In co-culture with mixed lymphocytes, mTOR-inhibited moDC exhibited a cytokine profile favoring a Th1 response with increased TNF and IFNG production and decreased IL10 concentrations. We conclude that mTOR inhibition in vitro promoted differentiation of inflammatory macrophages with reduced regulatory features and generation of Th1-favoring dendritic cells. These mechanisms could contribute to immune dysregulation in postpartum dairy cows.

## Introduction

Epidemiological studies have long established the association between alterations in metabolism and innate immune function and increased disease susceptibility in metabolically challenged cattle^1–3^, but the mechanisms behind this relationship are still under investigation. Morbidity and mortality are higher postpartum, a time of transient, but pronounced nutrient deficit, than at any other time in the lactation cycle, and cows show signs of systemic inflammation, greater susceptibility to infection, and reduced ability for efficient pathogen clearance compared with other stages of the lactation cycle^4,5^. The resulting health disorders in the early postpartum cow lead to increased economic losses and sustained use of antimicrobials in the dairy industry^6–8^. Postpartum cows show distinct changes in their immune cell phenotype and typical response to antigenic stimulation. In vitro-matured monocyte derived dendritic cells (moDC) from postpartum cows exhibited higher co-stimulatory activity and antigen presenting potential along with a reduced production of IL10 compared with moDC from late gestation cows^9^. Pro-inflammatory M1 macrophages (MPh) were found to infiltrate adipose tissue in diseased postpartum cows^10^. Although an increase in pro-inflammatory function is considered necessary postpartum to allow the cow to respond appropriately to infections, a dysregulation of this transition potentially induced by severe nutrient deficiencies might lead to an exaggerated immune response with detrimental consequences for the host^11^.

The mechanistic target of rapamycin (mTOR) is a conserved central intracellular pathway that senses nutrient balance and has emerged as a key orchestrator that integrates metabolic cues and reconciles these with immune activation^12,13^. At least two complexes exist for mTOR, mTORC1 and mTORC2. While mTORC1 integrates growth factor signaling, energy status (through AMP-activated protein kinase), and oxygen and amino acid availability^14^, mTORC2 is predominantly activated by growth factors^13^. Our previous work showed that the mTORC1 pathway is downregulated in muscle and adipose tissue postpartum, and both upstream AKT and mTORC1 kinase activity cannot be activated to the same degree postpartum compared with prepartum in vivo^15,16^. We have extended our characterization of the mTORC1 pathway to show reduced activation of this pathway in bovine peripheral blood mononuclear cells in the immediate postpartum period^17^ and that pathway activation can be partially rescued ex vivo by exogenous nutrient supply^18^. Interestingly, work in other species has shown that mTOR inhibition modulates pro-inflammatory responses of monocytes, MPh, and dendritic cells after lipopolysaccharide (LPS) stimulation in vitro^19^, and skews MPh to pro-inflammatory M1 cells^20,21^. Therefore, the observed characteristics of the immune cell phenotype of postpartum cows and the demonstrated reduction in mTOR activation may be linked. However, the possible role of the mTOR pathway in regulating MPh and moDC during the pronounced postpartum nutrient deficit in dairy cows has not been studied to date.

We hypothesized that the inhibition of mTOR during in vitro differentiation of bovine monocyte-derived MPh and moDC leads to alterations of phenotype and function that might be translatable to an exaggerated pro-inflammatory response during the postpartum period in vivo. Our objective was to evaluate the effects of incomplete and complete pharmacological mTOR pathway inhibition (1st and 2nd generation inhibitors, respectively) on MPh and dendritic cells, key regulators of the inflammatory response. Given the different roles that polarized MPh subsets are thought to have in vivo, we studied the effect of mTOR inhibition separately during differentiation of classically (M1) and alternatively (M2) activated MPh from circulating monocytes.

## Materials and methods

### Animals and blood sampling

All procedures were evaluated and approved by the Cornell University Institutional Animal Care and Use Committee (protocol no. 2019-0030) and all experiments were performed in accordance with relevant guidelines and regulations. We also complied with the ARRIVE guidelines^22–28^. We sampled multiparous, lactating animals (n = 12) between January and March of 2020. Average (± SD) parity, days since calving, and days pregnant were 2.7 ± 0.7, 253 ± 28 days, and 178 ± 22 days, respectively. Milk composite sample linear score of 2.1 ± 1.2 and lack of clinical signs indicated absence of mastitis. To select animals in positive energy balance and avoid sampling from immune activated animals, all cows had to lack clinical signs of other detectable diseases at time of sampling, had no record of any disease in the last 30 days and could not have received a vaccine within 30 d prior to the time of sampling. We collected all blood samples before the morning feeding (0700–0800 h) from the coccygeal vessels in an aseptic manner using a 20 g × 2.5 cm needle into sterile vacutainers containing either K_3_EDTA, 143 USP units of freeze-dried sodium heparin, or into vacutainers without additive for serum separation. Samples were transported immediately to the laboratory.

Blood was allowed to clot for 30 min at room temperature before harvesting serum by centrifugation at 2800 × *g* for 20 min at 4 °C. Whole EDTA blood was submitted to the Cornell University Animal Health Diagnostic Center Clinical Pathology Lab (Cornell University, Ithaca, NY, USA) for automated complete blood cell counts (Advia 2120, Siemens, Munich, Germany). Average ± SD blood cell counts for WBC, neutrophils, lymphocytes, and monocytes were 9.4 ± 5.0 × 10^3^/µL, 3.2 ± 0.7 × 10^3^/µL, 5.2 ± 4.3 × 10^3^/µL, and 0.5 ± 0.3 × 10^3^/µL, respectively.

### Cell separation and sorting

All primary antibodies are listed in Table 1. We obtained peripheral blood mononuclear cells (PBMC) from an average ± SD of 125 ± 15 mL of heparinized whole blood by density gradient separation as previously described^23^. Subsequently, we enriched CD14^+^ monocytes by magnetic activated cell sorting (MACS, all reagents Miltenyi Biotech, Bergisch Gladbach, Germany) as previously described^9^. Briefly, PBMC were labeled with a mouse anti-human monoclonal CD14 antibody (confirmed cross-reactive with bovine CD14 per manufacturer) coupled to paramagnetic beads and passed over a MACS separation column. Positively-selected enriched monocytes were counted, washed, and resuspended in complete medium (phenol-free RPMI 1640 with l-glutamine, 5% v/v FBS, 1× non-essential amino acids, and 1× insulin-transferrin-selenium) at 10^6^ cells/mL for in vitro cell culture to generate MPh and moDC in the presence of mTOR inhibitors as described below. CD14-depleted cells were counted, washed with PBS, and frozen at − 80 °C at 15 × 10^6^ cells per mL in freezing medium (45% v/v FBS, 9% v/v DMSO, 1% v/v HEPES) for later use in monocyte-derived dendritic cell (moDC) co-culture as autologous mixed lymphocytes. We determined purity and viability of CD14^+^ monocytes and CD14-depleted cells by flow cytometry. An aliquot of PBMC, CD14^+^ selected, and depleted cells were used to prepare cytospins (5 × 10^4^ cells diluted in 100 µL PBS) for differential cell counts performed by a board-certified clinical pathologist (Animal Health Diagnostic Center, Ithaca, NY). Successful cell separation with our protocol was confirmed with cytospin manual differential cell counts showing that CD14^+^ enriched cells were 91.0 ± 11.6% monocytes and CD14^+^ depleted cells were 86.5 ± 7.8% lymphocytes. Cell viability following Ficoll separation was 93.1 ± 7.8% for PBMC and cell viability following MACS separation was 96.2 ± 1.8% for CD14^+^ and 93.0 ± 2.5% for CD14^+^ depleted cells. Further flow cytometric characterization of the CD14^-^ cells showed an average (± SD) of 52.7 ± 9.3%, 20.7 ± 6.5%, 14.5 ± 6.4%, and 30.2 ± 10.4% of cells positive for MHCII, CD21, CD4, and CD3, respectively.

**Table 1:** Monoclonal antibodies used for flow cytometry, cell culture, and cytokine detection.

Specificity	Isotype	Clone	Conjugation	Source
CD3	IgG1	MM1A	–	Kingfisher Biotech
CD4	IgG2a	CC8	A647	Biorad
CD28	IgM	TE1A	–	Kingfisher Biotech
CD14	IgG1	CAM36A	–	Kingfisher Biotech
CD14^a^	IgG1	TUK4	MACS beads	Miltenyi Biotech
CD80	IgG1	ILA150	–	Biorad
CD163	IgG1	LND68A	–	Kingfisher Biotech
MHCII	IgG2a	TH14B	–	Kingfisher Biotech
IFNG	IgG1	CC302	FITC	Biorad
IFNG	IgG1	CC330	MicroPlex bead 35	Biorad
IFNG	IgG1	CC302	Biotin	Biorad
TNF	IgG1	197-1	MicroPlex Bead 32	Sipka et al.^29^
TNF	IgG1	65-2	Biotin	Sipka et al.^29^
IL10	IgG2b	CC318	MicroPlex Bead 33	Biorad
IL10^b^	IgG1	492-2	Biotin	Sipka et al.^29^

^a^Anti-human cross reactive with bovine

^b^Anti-equine cross reactive with bovine. All other antibodies are anti-bovine.

### mTOR inhibitor treatments

Rapamycin (RAPA, #9904, Cell Signaling Technology, Danvers, MA, USA) and Torin-1 (#14379, Cell Signaling Technology) inhibitors were resuspended in DMSO, diluted in complete RPMI, and for all treatments added to cell suspensions at a final concentration of 100 nM^24^.

### Generation of M1- and M2-polarized monocyte-derived macrophages (MPh)

Our objective was to study the effect of mTOR inhibition in polarized macrophage subsets. Cells differentiated in the presence of M1 polarization stimuli IFNG and CSF2, referred to as classically activated M1 MPh, are characterized by *NOS2* expression and a pro-inflammatory cytokine response. Cells differentiated in the presence of M2 polarization stimuli IL4 and CSF1, referred to as alternatively activated M2 MPh, are characterized by CD163 expression and a greater anti-inflammatory IL10 cytokine response. For the generation of M1 MPh, CD14^+^ monocytes were differentiated in complete medium with bovine recombinant IFNG (10 ng/mL) and CSF2 (20 ng/mL). To generate M2 MPh, bovine recombinant IL4 and CSF1 (both 20 ng/mL) were added to complete medium. Cells were cultured at 2.5 to 5 × 10^5^ cells/well in 48-well plates at 37 ºC and 5% CO_2_. After 2 days, differentiation medium was changed and RAPA, Torin-1, or DMSO vehicle control were added. Replicate treatments were cultured in parallel to allow for measurement of different outcomes, described below. One day after addition of inhibitor treatments, all cells were stimulated with LPS (100 ng/mL, *E. coli* O111:B4, Sigma–Aldrich, Burlington, MA, USA) for either 16 h (cell surface marker expression and cytokine production), 4 h (mRNA expression), or left as unstimulated controls. Culture plates were centrifuged (250 × *g*, 5 min) and supernatants were collected and stored at -80ºC until cytokine analysis. Cells were detached with Accutase® (Thermo-Fisher Scientific, Waltham, MA, USA) for 10 min (37 ºC, 5% CO_2_), washed with PBS, and immediately analyzed for surface maker expression. For rt-qPCR cells were lysed in 350 µL RLT (Qiagen, Hilden, Germany) plus 0.1% v/v ß-mercaptoethanol and stored at – 80 ºC until RNA isolation.

### Monocyte-derived dendritic cells (moDC) and mixed lymphocyte reaction (MLR) co-culture

We were interested in studying the effect of mTOR inhibition on both naïve and mature moDC as well as on the effect of mTOR inhibited mature moDC on T-cell IFNG response. To generate naïve moDC, CD14^+^ monocytes were cultured in complete medium plus 20 ng/mL recombinant bovine CSF2 and IL4 as previously described^25^. Cells were cultured at 2.5 to 5 × 10^5^ cells/well in 48-well culture plates at 37 ºC and 5% CO_2_. On day 3, differentiation medium was changed and RAPA, Torin-1, or DMSO vehicle control were added with fresh medium. Replicate treatments were cultured in parallel to allow for measurement of different outcomes.

For cell surface marker expression, cytokine production, and gene expression profiles, culture medium was changed to inhibitor-free complete medium after 4 days of culture with inhibitor treatments, and moDC were matured for 16 h (surface marker, protein secretion), or 4 h (gene expression profiles) with LPS (100 ng/mL) or left as naïve moDC, and samples harvested as described above.

To study the effect of mTOR inhibited mature moDC on T-cell activation, we used co-cultures with the CD14-depleted cell fraction of PBMC from the same individual (mixed lymphocytes) in an autologous mixed lymphocyte reaction (MLR). Culture medium was changed to inhibitor-free complete medium after 4 days of culture with inhibitor treatments, and moDC were matured for 24 h with UV irradiated *E. coli* strain ECC-Z at a multiplicity of infection of 10 as previously described^25^ or left as naïve cells. Autologous MLR were thawed and incubated overnight in complete medium. Mature moDC and MLR were co-cultured at a ratio of 1:5^26–28^ in the presence of anti-bovine CD3 and anti-bovine CD28 (Table 1) at 1 µg/mL for 24 h. Then, supernatants were harvested and stored at – 80 ºC until cytokine analysis.

We used parallel moDC-MLR co-cultures to measure the intracellular IFNG production of T-cells by adding brefeldin A (500 ng/mL) in addition to anti-bovine CD3 and anti-bovine CD28 for 6 h. Monocultures of MLR were incubated in parallel and stimulated with 10 ng/mL PMA and 100 ng/mL ionomycin as positive control or left unstimulated as negative control. Cells were stained with fixable LIVE/DEAD® Near-IR Dead Cell Stain Kit (Invitrogen, Thermo Fisher Scientific), fixed with 2% v/v para-formaldehyde in PBS for 10 min, washed, and stored at 4 ºC until antibody labeling and flow cytometry.

### Membrane and intracellular immune fluorescence

We performed membrane immune fluorescence for CD14 expression on PBMC, CD14^+^ selected and depleted cells, CD163 and MHCII on M1 and M2 MPh, CD80 and MHCII on moDC, and for CD4 expression on autologous mixed lymphocytes to describe cell lineage. Cells were washed in FACS buffer (0.05% w/v sodium azide and 0.5% w/v BSA in PBS), pelleted, and incubated with primary antibodies for 30 min at 4 ºC. Then, cells were washed twice and incubated with FITC-conjugated goat pAb: anti-mouse IgG1 (Bio-Rad Laboratories) diluted in FACS buffer at 1:100 for 30 min at 4 °C and washed twice with FACS buffer. Samples were transferred to microfuge vials containing 100 μL propidium iodide (PI; 20 μg/mL), and immediately analyzed by flow cytometry. Intracellular fluorescence was performed with fixed moDC-MLR co-cultures after membrane immune fluorescence labeling was performed as described above. Samples were analyzed using an Attune acoustic focusing flow cytometer (Thermo Fisher Scientific) or, for intracellular fluorescence, a FACS Canto II flow cytometer (BD Biosciences). Doublets were excluded based on forward scatter (FSC) height and FSC area. Dead cells were excluded by gating on PI-negative events (unfixed cells) or on LIVE/DEAD Near-IR negative events (fixed cells). Cell morphology gates were drawn in forward and side scatter and 10,000 events were acquired for each measurement. Mean fluorescence intensity was corrected for background from bioparticles and loading dye, or background fluorescence of unstained or secondary controls, respectively. Data were analyzed using FlowJo V10 software (BD Biosciences, San Jose, CA).

### Phagocytosis and oxidative burst assays

Phagocytosis and oxidative burst of M1 and M2 MPh were analyzed in duplicate after 2 days of culture with inhibitor treatments. For measurement of phagocytosis, we first sonicated pHrodo bioparticles (pHrodo Green Zymosan Bioparticles Conjugate, Invitrogen, Carlsbad, CA), diluted these in 1X HEPES buffer (pH, 7.4), and opsonized with 1% v/v autologous serum for 1 h at 37 °C. Cells were incubated with pHrodo bioparticles or left as unstimulated control for 1 h at 37 °C.

For measurement of oxidative burst, we incubated cells with 10 µM H_2_DCFDA fluorescein loading dye (Invitrogen, Carlsbad, CA) for the detection of reactive oxygen species or left unstained as background control for 15 min at 37 °C. Subsequently, cells were stimulated with PMA at final concentrations of 0, 10, and 25 ng/mL, or left as unstimulated control for 15 min at 37 °C. Following incubation, cells were placed on ice, mixed with 100 μL propidium iodide (20 μg/mL) to assess cell viability, and analyzed by flow cytometry.

### Cytokine production and reverse-transcription quantitative PCR

Culture supernatants were used to quantify the concentration of IL10, TNF, and IFNG in a bead-based multiplex assay as described^29^.

For RNA extraction, cell lysates in RLT buffer were rapidly thawed at 37 °C for 10 min and homogenized using a spin column (QIAshredder, Qiagen, Venlo, Netherlands). RNA was extracted using the RNeasy Plus Mini Kit (Qiagen) and characterized and quantified using a NanoDrop One spectrophotometer (Thermo Fisher Scientific). Samples with an optical density 260:280 ratio ≥ 1.8 but ≤ 2.2 were processed further. A volume containing 0.25 μg of RNA was reverse transcribed (SuperScript IV First-Strand cDNA Synthesis, Life Technologies, Carlsbad, CA, USA) and stored at − 20 °C. A pool of cDNA from all samples served as the calibrator sample. Bovine-specific primer probe sets (TaqMan Gene Expression Assays, Applied BioSystems, Thermo Fisher Scientific) with exon spanning probes were purchased for genes of interest [*IL12A* (p35): Bt03213922, *IL12B* (p40): Bt03213923, *IL1B*: Bt03212741, *IL6*: Bt03211905, *NOS2*: Bt03249586, *ARG2*: Bt03219827] and 2 reference genes (*RPLP0*: Bt03218087, *TBP*: Bt03241946). Both reference genes were previously shown to be stably expressed in bovine leukocytes and used in a similar experimental setup^21^. Real-time reverse-transcription quantitative PCR was performed in triplicate using a 1:3 (moDC) or 1:6 (M1 and M2 Mph) dilution of cDNA at 20% v/v of the final 10-μL reaction volume using TaqMan Gene Expression Master Mix (Applied BioSystems, Thermo Fisher Scientific) in a QuantStudio 12 K Flex Real-Time PCR System (Applied BioSystems, Thermo Fisher Scientific). Run conditions for all genes of interest were 1 cycle at 50 °C for 2 min, 1 cycle at 95 °C for 10 min, followed by 40 cycles of 95 °C for 15 s and 60 °C for 1 min. Raw data were analyzed using the comparative quantification method (ΔΔCt method, QuantStudio 12 K Flex Software, v1.2.2) and expressed as relative quantity (RQ = 2^−ΔΔCt^) before export for statistical analysis.

### Analytical approach

Sample size was based on the expected difference in IFNG production of co-culturing MLR with rapamycin/Torin-1-conditioned moDC. Our conservative expectation was to find an effect at least as large as 50% of the effect of mTOR inhibition on increased IFNG production as a marker for Th1-cell response performed in human cells described previously^26^. The sample size of 12 animals is based on an expected difference of 25 ng/mL (90 vs. 115 ng/mL) with a conservative SD of 20 ng/mL of IFNG with probabilities of type I and II error set to 0.05 (alpha = 0.05; power = 0.95), using a statistical software program (GPower v. 3.1.9.2^30^) with assumed correlation between samples within a cow of 0.5.

All data were analyzed in the statistical software package SAS (v. 9.4, SAS Institute Inc., Cary, NC, USA). Data describing the study population and cell subsets were summarized in PROC UNIVARIATE. All other data were analyzed using mixed effects ANOVA in PROC MIXED. Models were corrected for parity and DIM and included a random effect of cow. Outlier diagnostics were performed as defined a priori with the INFLUENCE statement and removed if Cook’s D was > 0.5. Model residuals were visually inspected for normality and homoscedasticity and data log transformed to fulfill model assumptions as indicated. Results are presented as least squares means (LSM) and standard error (SE), or geometric LSM and backtransformed 95% confidence interval (CI) unless otherwise specified. Graphs were produced in GraphPad Prism (v. 9.3.1, La Jolla, CA).

For all models, pairwise comparisons within fixed effects were performed if the F-test for the effect yielded a *P* ≤ 0.05. Multiple comparisons corrections were performed with Bonferroni or Tukey’s correction as indicated.

Differences between polarized MPh surface markers, gene expression, and cytokine production with and without LPS stimulation were analyzed in a model that included fixed effect of cell type, stimulus, and cell type × stimulus interaction. Pairwise comparisons were applied to LSM to test the effect of stimulus within and between MPh with Bonferroni correction. Difference in phagocytosis was analyzed in a model with fixed effect of MPh type and differences in oxidative burst were analyzed in a model with fixed effect of MPh type, PMA dose, and cell type × PMA interaction; pairwise comparisons were corrected using Tukey’s posthoc procedure. Data for phagocytosis, gene expression, and cytokine production were log transformed before analysis. Differences between naïve and LPS-matured moDC, and between naïve and *E. coli*-matured moDC in MLR co-cultures, were analyzed in a model with fixed effect of LPS or *E. coli* treatment, respectively. Gene expression and cytokine production data were log transformed. Treatment differences in polarized MPh and moDC phenotype and function were analyzed separately by stimulation (without or with LPS or PMA in MPh), or maturity (without or with LPS or *E. coli* in naïve and mature moDC, respectively) before analysis. Surface marker, phagocytosis, oxidative burst, and cytokine production data were all expressed relative to CTRL and log transformed. Gene expression data were analyzed as the fold change (FC) from CTRL and log transformed. All data were analyzed with fixed effect of inhibitor treatment to test the effect of RAPA vs. TORIN-1 and pairwise comparison of RAPA vs. 0 and TORIN-1 vs. 0 in LSMEANS, all with Bonferroni correction. Gene expression data are presented as FC from CTRL, and all other data are presented as the relative change from CTRL.

## Results

We investigated the effect of pharmacological mTOR inhibition during differentiation of monocytes to M1 (IFNG, CSF2) and M2 (IL4, CSF1) MPh, as well as moDC (IL4, CSF2) to simulate prolonged cellular nutrient deficit as experienced by the transition dairy cow. Generated M1 and M2 MPh were clearly distinct in phenotype and function, with differences in expression of MHCII, CD163, and *NOS2* expression (Supplementary Fig. 1), as well as phagocytosis, oxidative burst, and cytokine mRNA expression (Supplementary Fig. 2).

### Inhibition of mTOR generated inflammatory M1 with decreased phagocytosis and IL10 production

**Figure 1:**
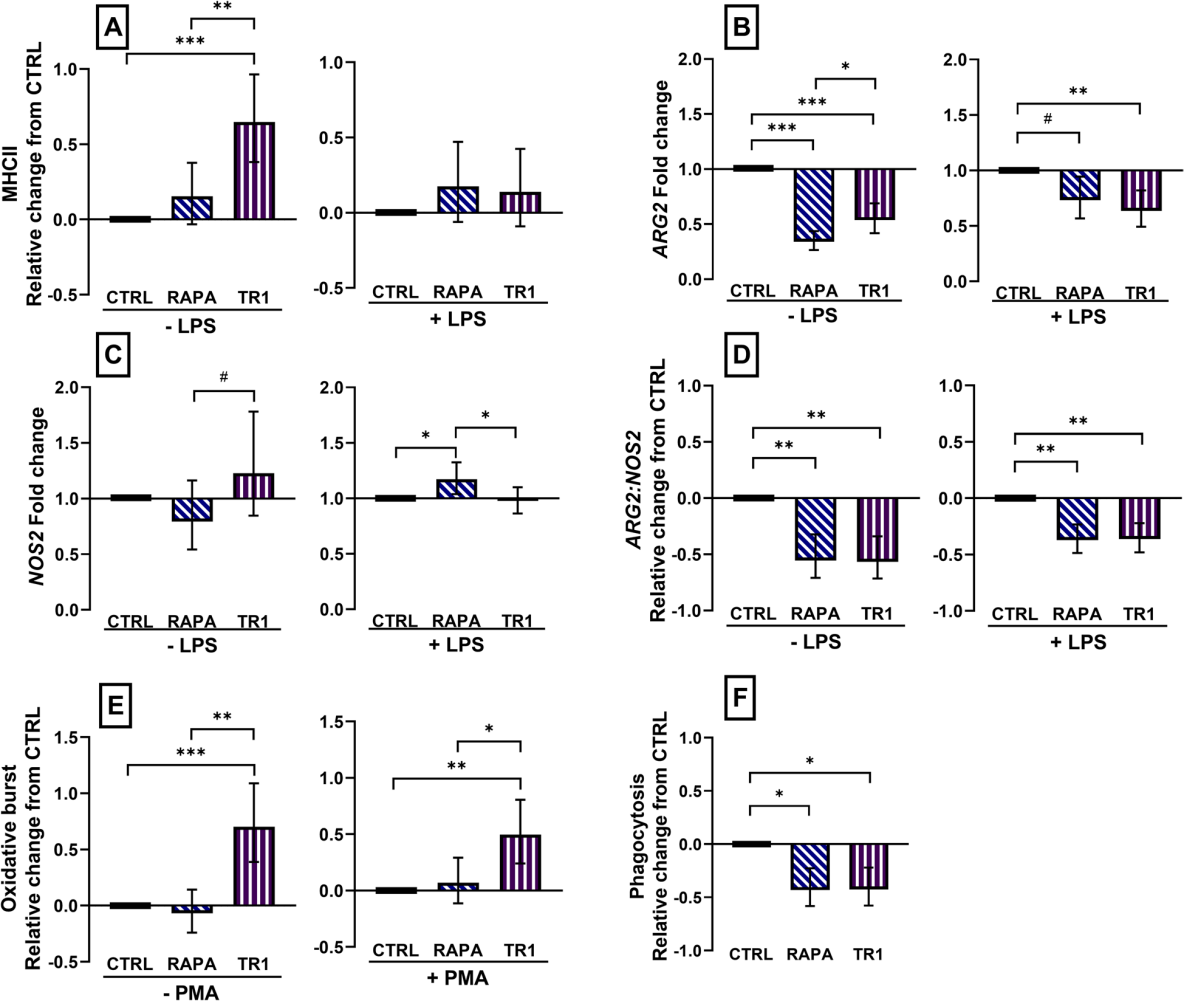
Changes in surface maker expression, mRNA abundance, oxidative burst, and phagocytosis in M1 macrophages polarized for 3 days with 10 ng/mL IFNG and 20 ng/mL CSF2 and concurrently treated with vehicle (CTRL), or inhibitors rapamycin (RAPA) or Torin-1 (T1) at 100 nM. Cells were incubated with vehicle (− LPS) or stimulated with 100 nM LPS (+LPS) for 16 h to measure change in MHCII expression (**A**), or 4 h to measure changes in mRNA abundance of *arginase 2* (*ARG2,* **B**) and *inducible nitric oxide synthase* (*NOS2,* **C**) as well as their ratio (**D**). Cells were incubated with 10 µM H_2_DCFDA fluorescein loading dye for 15 min and stimulated with phorbol myristate acetate (PMA) at 0 (− PMA), or 25 (+ PMA) ng/mL for 15 min to measure oxidative burst (**E**) or with pHrodo bioparticles for 1 h to measure cellular phagocytosis (**F**). Data were expressed relative to CTRL and presented as the relative change from CTRL in unstimulated and stimulated cells for MHCII, *ARG2:NOS2*, phagocytosis, and oxidative burst. mRNA abundance was expressed as the fold change from CTRL in unstimulated and stimulated cells. Data from cell isolation of 12 cows are presented as the geometric mean and backtransformed 95% confidence interval. ANOVA tests between CTRL and inhibitors, and between inhibitors are presented with Bonferroni-adjustment for multiple comparisons: *P* ≤ 0.10 (^#^), *P* ≤ 0.05 (*), *P* ≤ 0.01 (**), *P* ≤ 0.0001 (***).

We found distinct effects of both mTOR inhibitors on the phenotype of classically activated M1 MPh (with IFNG) that were additionally stimulated with LPS or left unstimulated. Surface MHCII expression was increased (*P* < 0.0001) in Torin-1-treated, but not (*P* = 0.30) RAPA-treated unstimulated M1 cells; no effect (*P* > 0.70) of inhibitor treatment on MHCII expression was found in LPS-stimulated cells (Fig. 1A). Both RAPA and Torin-1-treated cells had decreased (*P* ≤ 0.06) *ARG2* expression in unstimulated and stimulated M1 MPh (Fig. 1B).

Expression of *NOS2* increased (*P* ≤ 0.04) in RAPA-treated and LPS-simulated cells and the ratio of *ARG2:NOS2* was decreased (*P* < 0.01) in both RAPA and Torin-1 for unstimulated and stimulated cells (Fig. 1C, D). As expected, expression of CD163 was not detected or was minimal in M1 MPh and was therefore not analyzed further.

The inhibition of mTOR during differentiation also affected intracellular killing potential of M1 MPh. Phagocytosis was decreased by both RAPA (*P* = 0.01) and Torin-1 (*P* = 0.01) treatment (Fig. 1). Moreover, oxidative burst was increased (*P* < 0.01) in Torin-1 treated cells, but not in RAPA treated cells (*P* > 0.99) (Fig. 1E).

**Figure 2:**
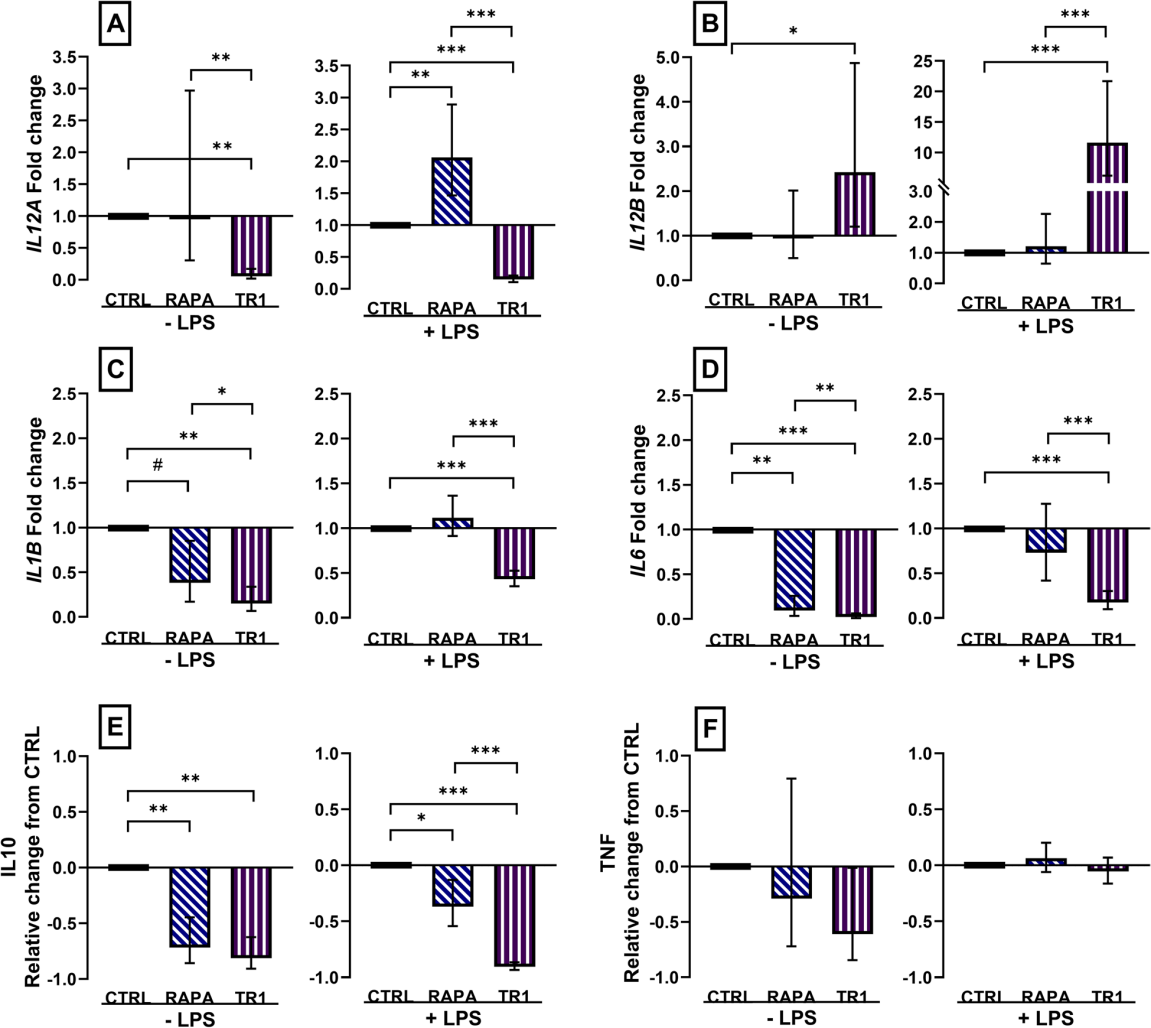
Changes in cytokine mRNA abundance and production in M1 macrophages polarized for 3 days with 10 ng/mL IFNG and 20 ng/mL CSF2 and concurrently treated with vehicle (CTRL), or inhibitors rapamycin (RAPA) or Torin-1 (TR1) at 100 nM. Cells were incubated with vehicle (− LPS) or stimulated with 100 nM LPS (+ LPS) for 4 h to measure changes in mRNA abundance of *IL12A*, *IL12B*, *IL1B*, and *IL6* gene expression (**A**–**D**) or for 16 h to measure production of IL10 and TNF protein (**E**, **F**). mRNA abundance was expressed as the fold change from CTRL in unstimulated and stimulated cells. Data were expressed relative to CTRL and presented as the relative change from CTRL in unstimulated and stimulated cells for cytokine production. Data from cell isolation of 12 cows are presented as the geometric mean and backtransformed 95% confidence interval. ANOVA tests between CTRL and inhibitors, and between inhibitors are presented with Bonferroni-adjustment for multiple comparisons: *P* ≤ 0.10 (^#^), *P* ≤ 0.05 (*), *P* ≤ 0.01 (**), *P* ≤ 0.0001 (***).

The two mTOR inhibitors also affected mRNA expression of the two IL12 subunits. Expression of *IL12A* was decreased (*P* ≤ 0.01) in Torin-1-treated unstimulated and stimulated M1 MPh but increased by two-fold (*P* = 0.0009) in RAPA-treated stimulated cells (Fig. 2A). Expression of *IL12B* was increased more than two-fold (*P* ≤ 0.05) in Torin-1-treated unstimulated and more than ten-fold in stimulated M1 MPh (Fig. 2B). Treatment with either inhibitor decreased *IL1B* and *IL6* expression (*P* ≤ 0.07) in unstimulated cells, but only Torin-1 decreased (*P* < 0.0001) cytokine expression in LPS-stimulated M1 MPh (Fig. 2C, D). Importantly, the secretion of IL10 in culture medium was decreased for both RAPA and Torin-1-treated naïve and stimulated M1 MPh (*P* ≤ 0.02). Inhibitor treatment did not change (*P* ≥ 0.14) the secretion of TNF from cells (Fig. 2E, F). Overall, these changes in cytokine expression profile show a selective effect of mTOR inhibition on the pro-inflammatory cytokine profile of M1 MPh, but a consistent decrease in the anti-inflammatory cytokine IL10.

### Inhibition of mTOR interfered with the differentiation of M2 macrophages

**Figure 3:**
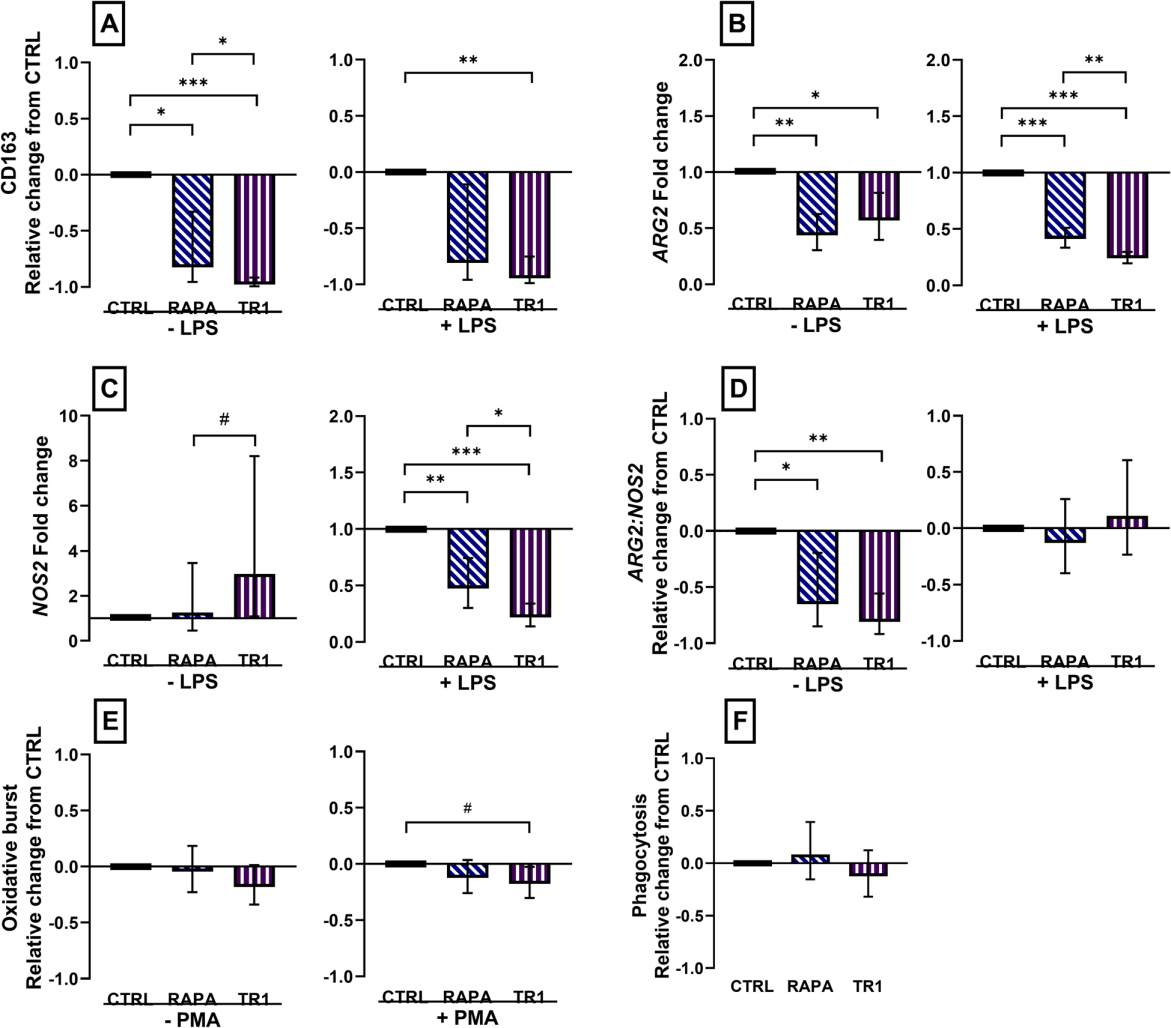
Changes in surface maker expression, mRNA abundance, oxidative burst, and phagocytosis in M2 macrophages polarized for 3 days with 20 ng/mL IL4 and 20 ng/mL CSF1 and concurrently treated with vehicle (CTRL), or inhibitors rapamycin (RAPA) or Torin-1 (TR1) at 100 nM. Cells were incubated with vehicle (− LPS) or stimulated with 100 nM LPS (+ LPS) for 16 h to measure change in CD163 expression (**A**), or 4 h to measure changes in mRNA abundance of *arginase 2* (*ARG2,* **B**) and *inducible nitric oxide synthase* (*NOS2,* **C**) and their ratio (**D**). Cells were incubated with 10 µM H_2_DCFDA fluorescein loading dye for 15 min and stimulated with phorbol myristate acetate (PMA) at 0 (− PMA), or 25 (+ PMA) ng/mL for 15 min to measure oxidative burst (**E**), or for 1 h with pHrodo bioparticles to measure cellular phagocytosis (**F**). Data were expressed relative to CTRL and presented as the relative change from CTRL in unstimulated and stimulated cells for CD163, *ARG2:NOS2*, phagocytosis, and oxidative burst. mRNA abundance was expressed as the fold change from CTRL in unstimulated and stimulated cells. Data from cell isolation of 12 cows are presented as the geometric mean and backtransformed 95% confidence interval. ANOVA tests between CTRL and inhibitors, and between inhibitors are presented with Bonferroni-adjustment for multiple comparisons: *P* ≤ 0.10 (^#^), *P* ≤ 0.05 (*), *P* ≤ 0.01 (**), *P* ≤ 0.0001 (***).

Next, we examined the effect of mTOR inhibitors during polarization on M2 MPh that were left unstimulated or stimulated with LPS. Expression of the M2 marker CD163 was decreased (*P* ≤ 0.04) by RAPA and Torin-1 in unstimulated M2 MPh. Only Torin-1 significantly decreased (*P* = 0.003) CD163 expression in LPS-stimulated M2 (Fig. 3A). Expression of *ARG2* was decreased (*P* ≤ 0.01) in both RAPA and Torin-1-treated unstimulated and LPS-stimulated cells, whereas expression of *NOS2* was decreased (*P* ≤ 0.009) in both RAPA and Torin-1-treated stimulated cells (Fig. 3B, C). Changes in expression of *ARG2* and *NOS2* resulted in decreased (*P* ≤ 0.05) ratio of *ARG2:NOS2* in RAPA- and Torin-1-treated unstimulated M2, but the effect did not differ (*P* = 0.36) by inhibitor treatment, and inhibitor treatment did not change (*P* ≥ 0.99) the ratio in stimulated M2 (Fig. 3D).

Different from M1 MPh, we only observed moderate changes in functional assays in response to mTOR inhibition in M2 MPh. Compared with CTRL, RAPA treatment did not affect (*P* ≥ 0.34) oxidative burst, but Torin-1 decreased (*P* = 0.08) the oxidative burst response (Fig. 3E). No effect (*P* ≥ 0.81) of inhibitor treatment on phagocytosis was found (Fig. 3F).

**Figure 4:**
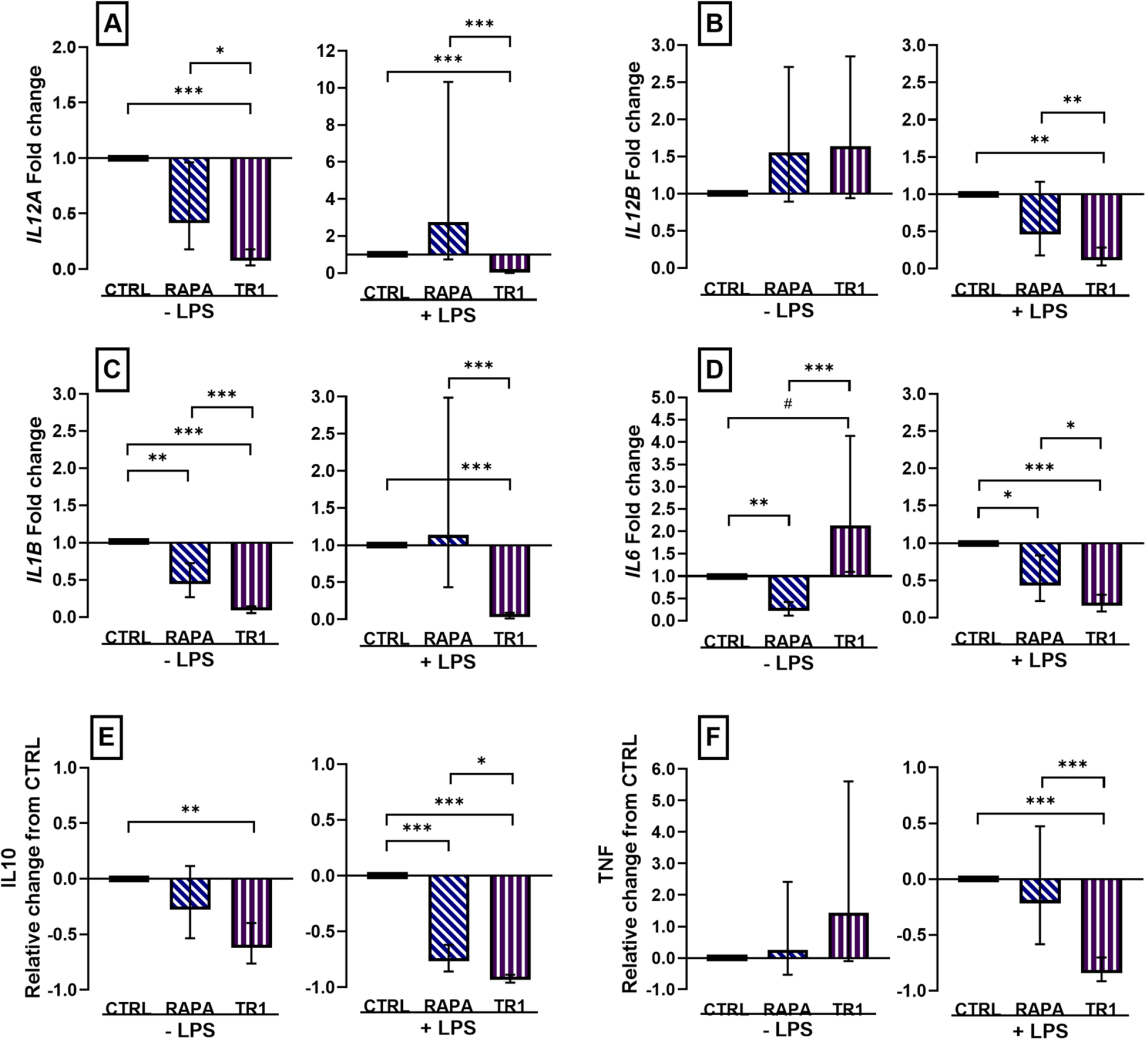
Changes in cytokine mRNA abundance and production in M2 macrophages polarized for 3 days with 20 ng/mL IL4 and 20 ng/mL CSF1 and concurrently treated with vehicle (CTRL), or inhibitors rapamycin (RAPA) or Torin-1 (TR1) at 100 nM. Cells were incubated with vehicle (− LPS) or stimulated with 100 ng/mL LPS (+ LPS) for 4 h to measure changes in mRNA abundance of *IL12A*, *IL12B*, *IL1B,* and *IL6 *(**A**–**D**), or for 16 h to measure production of IL10 and TNF protein (**E**, **F**). mRNA abundance was expressed as the fold change from CTRL in unstimulated and stimulated cells. Data from cell isolation of 12 cows were expressed relative to CTRL and presented as the relative change from CTRL in unstimulated and stimulated cells for cytokine production. Data are presented as the geometric mean and backtransformed 95% confidence interval. ANOVA tests between CTRL and inhibitors, and between inhibitors are presented with Bonferroni-adjustment for multiple comparisons: *P* ≤ 0.10 (^#^), *P* ≤ 0.05 (*), *P* ≤ 0.01 (**), *P* ≤ 0.0001 (***).

Unlike the opposing effects that mTOR inhibition had on M1, expression of *IL12A* and *IL12B* was only affected by Torin-1 in M2 MPh. Expression of *IL12A* was decreased (*P* < 0.0001) in Torin-1-treated unstimulated and LPS-stimulated M2 whereas LPS-stimulated Torin-1-treated M2 cells had decreased (*P* = 0.0009) *IL12B* (Fig. 4A, B). Expression of *IL1B* was decreased (*P* < 0.0001) in both unstimulated and stimulated Torin-1-treated M2 and decreased (*P* ≤ 0.009) in RAPA and Torin-1-treated M2 MPh (Fig. 4C). Treatment with RAPA during polarization was associated with decreased (*P* ≤ 0.05) *IL6* expression in unstimulated and stimulated cells, but Torin-1 increased *IL6* (*P* = 0.09) in unstimulated M2 cells whereas it decreased *IL6* (*P* = 0.03) in LPS-stimulated cells (Fig. 4D). Concentration of IL10 was decreased (*P* < 0.001) in cell culture supernatant from unstimulated and stimulated cells that were treated with Torin-1 during polarization, and to a smaller degree in LPS-stimulated cells treated with RAPA (*P* < 0.0001) (Fig. 4E). Concentration of TNF was decreased (*P* < 0.0001) in cell culture supernatant from stimulated cells that were treated with Torin-1 during polarization (Fig. 4F). Overall, mTOR inhibition during differentiation of M2 MPh resulted in decreased expression of pro-inflammatory cytokines except for *IL12B* and *IL6* in unstimulated M2, and the same consistent decrease in IL10 production was observed as in M1 MPh under mTOR inhibition.

### Inhibition of mTOR during moDC differentiation increased co-stimulatory potential and favored a Th1 cytokine profile

As a third monocyte-derived cell population we investigated how treatment with mTOR inhibitors affected the differentiation of moDC, their response when matured with LPS, and cytokine production when co-cultured with an autologous MLR.

**Figure 5:**
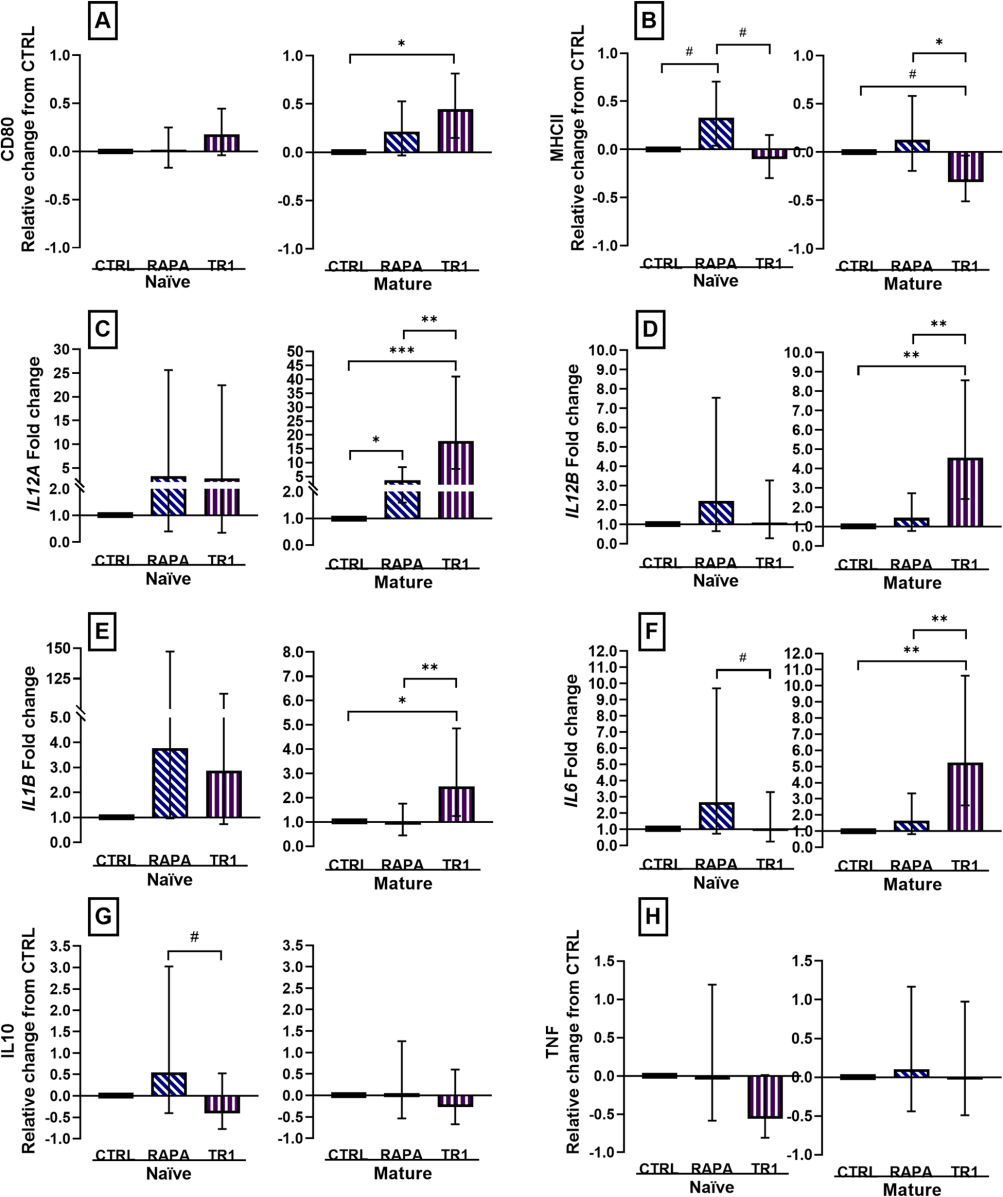
Changes in surface maker expression, mRNA abundance, and cytokine production in moDC after differentiation for 7 days with 20 ng/mL CSF2 and IL4 and concurrently treated with vehicle (CTRL), or inhibitors rapamycin (RAPA) or Torin-1 (TR1) at 100 nM during the last 4 days of differentiation. Naïve moDC (Naïve) were matured with 100 ng/mL LPS (Mature) for 16 h to characterize surface markers CD80 (**A**) and MHCII (**B**) and measure cytokine production of IL10 (**G**) and TNF (**H**) protein, and for 4 h to quantify mRNA abundance of *IL12A, IL12B, IL1B*, and *IL6* (**C**–**F**). Data were expressed relative to CTRL and presented as relative change from CTRL in naïve and mature moDC for surface marker expression and cytokine production. mRNA abundance was expressed as the fold change from CTRL in naïve and mature moDC. Data from cell isolation of 12 cows are presented as geometric mean and backtransformed 95% confidence interval. ANOVA tests between CTRL and inhibitors, and between inhibitors are presented with Bonferroni-adjustment for multiple comparisons: *P* ≤ 0.10 (^#^), *P* ≤ 0.05 (*), *P* ≤ 0.01 (**), *P* ≤ 0.0001 (***).

First, we were interested in examining possible changes in naïve moDC to characterize the potential effects of mTOR inhibition on DC prior to antigen encounter, with results indicating no significant changes in phenotype (Fig. 5) with the exception of greater surface MHCII expression (*P* = 0.08) in RAPA-treated naïve moDC.

Next, we analyzed the effects of the inhibitors on mature moDC. The LPS-matured moDC were characterized by up-regulation of antigen-presenting and co-stimulatory molecules needed to activate T cells and by their cytokine profile (Supplemental Fig. 3). Surface CD80 expression was increased (*P* = 0.01) in Torin-1-treated mature moDC (Fig. 5A) and MHCII tended to decrease (*P* = 0.10) in Torin-1-treated mature moDC (Fig. 5B). Treatment with mTOR-inhibitors during moDC differentiation changed the expression profile of selected cytokines in mature moDC (Fig. 5C–F): both Torin-1 (*P* = 0.0006) and RAPA (*P* = 0.02) increased expression of *IL12A*, only Torin-1 increased expression of *IL12B*, *IL1B* and *IL6*, whereas secretion of IL10 and TNF in cell culture supernatants of mature moDC was not altered (*P* ≥ 0.16) by inhibitor treatment (Fig. 5G, H). In summary, mTOR inhibition with Torin-1 altered moDC surface marker expression, increased expression of pro-inflammatory cytokines, but did not affect the concentration of anti-inflammatory IL10, whereas RAPA showed only minor effects.

**Figure 6:**
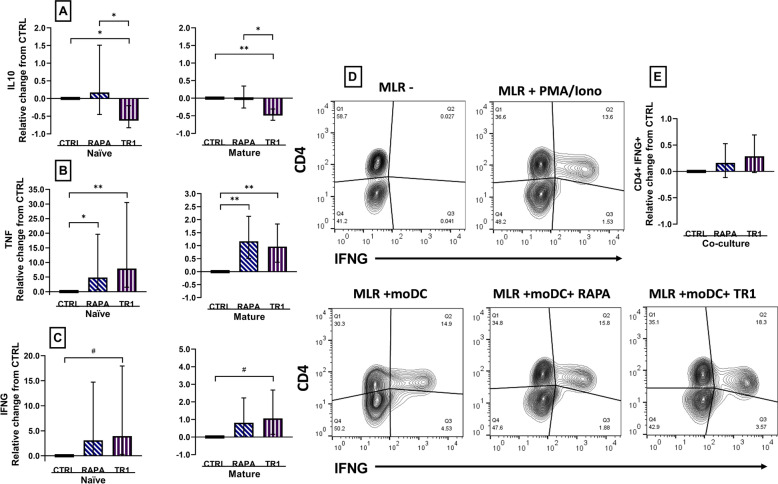
Changes in cytokine concentration of IL10 (**A**), TNF (**B**), and IFNG (**C**) measured in supernatants from moDC that were concurrently treated with vehicle (CTRL), or inhibitors rapamycin (RAPA) or Torin-1 (TR1) at 100 nM during the last 4 days of differentiation before maturation with *E. coli* (Mature), co-cultured with mixed lymphocyte reaction (MLR), and T-cell activated with anti CD3/CD28. Naïve moDC that were not matured with *E. coli* were co-cultured and activated in parallel (Naïve). (**D**) Intracellular IFNG expression in CD4^+^ MLR was analyzed via flow cytometry of MLR co-cultured for 1 day with in vitro-generated moDC (7-day differentiation protocol with 20 ng/mL CSF2 and IL4, matured for 1 day with *E. coli*) before activation with anti CD3/CD28 and concurrent incubation with brefeldin A for 6 h. Cells were labeled with anti-bovine CD4 FTIC-conjugated antibodies and after fixation and permeabilization with anti-bovine IFNG A647. Contour plots show fluorescence for CD4-FITC and IFNG A647 of MLR negative controls (w/o moDC), positive controls (stimulated with phorbol myristate acetate (PMA) and ionomycin (Iono), and co-cultures of MLR with moDC treated with vehicle, RAPA or Torin-1. (**E**) Mean fluorescence intensity (MFI) of IFNG staining in CD4+ MLR relative to CRTL. Data from cell isolation of 12 cows were expressed relative to CTRL and presented as relative change from CTRL in naïve and mature moDC. Data are presented as geometric mean and backtransformed 95% confidence interval. ANOVA tests between CTRL and inhibitors, and between inhibitors are presented with Bonferroni-adjustment for multiple comparisons: *P* ≤ 0.10 (^#^), *P* ≤ 0.05 (*), *P* ≤ 0.01 (**), *P* ≤ 0.0001 (***).

Mature mTOR inhibitor-treated moDC were co-cultured with autologous MLR to study changes in Th1-derived IFNG production as an indirect assessment of moDC IL12 cytokine production. Concentration of IFNG was 20-fold greater (*P* < 0.0001) in co-cultures with mature moDC [3.0 (1.5–6.0) vs. 69 (36–131) ng/mL in naïve and mature moDC, respectively]. Concentration of IFNG was increased (*P* ≤ 0.07) in co-cultures of naïve and mature moDC that were treated with Torin-1 (Fig. 6C), but no treatment differences were found in intracellular IFNG measurements of CD4^+^ cells (Fig. 6D, E). As described above, the sample size of 12 individual cows for this study was based on an expected difference of 25 ng/mL and a SD of 20 ng/mL IFNG between treatments. Concentration of IFNG in mature co-cultures averaged (± SD) 89.0 ± 46.8, 134 ± 49, and 147 ± 40 ng/mL for CTRL, RAPA, and Torin-1 respectively, showing that both magnitude of difference between the mTOR inhibitor treatments and CTRL, as well as SD were about twice as high as described above. Regarding the concentration of other cytokines of interest in co-culture, IL10 was decreased (*P* ≤ 0.04) in supernatants of Torin-1-treated naïve and mature moDC, whereas TNF concentrations increased (*P* ≤ 0.03) in co-cultures of both naïve and mature moDC that were treated with RAPA or Torin-1 (Fig. 6A, B).

## Discussion

Our objective in this study was to investigate the role of mTOR pathway inhibition on key regulators of the bovine innate and early adaptive immune response. We simulated nutrient deficit-induced mTOR suppression by using pharmacological mTOR inhibition in an in vitro model. Postpartum dairy cows undergo a time of pronounced nutrient deficit with decreased availability of energy and essential amino acids, as well as reduced circulating concentrations of insulin and IGF1^31–33^. At the same time, these animals often display immune dysfunction that is characterized by a heightened inflammatory response and decreased ability to clear pathogens^7,34^. Several studies in cattle have shown an association of the severity of negative nutrient balance and immune dysfunction^35,36^, but the causal mechanisms are not well understood to date. In other species, research has shown that the mammalian metabolic status is integrally linked with immune cell function, and that the mTOR pathway is a key integrator of nutrient-sensing and immune activation^37^. Our previous work indicates a reduced activation of components of the AKT-mTOR pathway in muscle, adipose tissue and peripheral blood mononuclear cells harvested from postpartum dairy cows^15–17,38^.

The observed net effect of mTOR inhibition on M1 MPh in our model suggests a selective regulation of M1 features with potentially decreased pathogen clearance and activation of other phagocytic cells, but concurrent increase in production of potentially damaging free radicals. Activation of M1 MPh typically occurs upon encountering a pathogen, whereas M2 MPh play a role in the resolution of this response^39,40^. Short-term (48 h) addition of RAPA at the end of a differentiation and polarization protocol shifted M2 polarized human MPh towards an M1-like profile, and increased cell apoptosis^20^. In our LPS-stimulated bovine M1 MPh model, long-term exposure to mTOR inhibitors during cell differentiation appeared to selectively affect the pro-inflammatory M1 response to stimulation. This was represented by a decrease of the *ARG2:NOS2* ratio (RAPA, Torin-1)*,* increase of *IL12A* (RAPA) or *IL12B* (Torin-1)*,* as well as increased ROS production and decreased anti-inflammatory IL10 production (RAPA, Torin-1). This is in line with other findings in murine models where mTOR inhibition induced an M1 phenotype and genetic deletion of mTORC1 enhanced pro-inflammatory cytokines^41,42^. In contrast to those findings, we observed that both inhibitors decreased phagocytosis of bovine M1, and treatment with Torin-1 specifically decreased *IL6,* and *IL1B* expression in LPS-stimulated M1.

Furthermore, mTOR inhibition impaired M2 polarization, measured by lower production of IL10, drastically reduced expression of CD163 and *ARG2*, which are hallmark features of this regulatory cell type^43^. Polarization towards an M2 phenotype is mTOR-dependent^44^, and inhibition of this pathway during M2 polarization may reduce survival^20^. This shift toward an M1 phenotype is consistent with changes observed in human MPh^20^. The results from our model suggest that mTOR inhibition might prevent the IL4 driven differentiation of M2 which could impair restoration of homeostasis and extend the pro-inflammatory response. In response to LPS stimulation however, we demonstrated that M2 differentiated under mTOR inhibition were clearly different in response to M1 MPh and showed decreased expression of pro-inflammatory cytokines (*IL12A, IL12B, IL1β, IL6*) as well as TNF concentration in addition to drastically decreasing CD163 surface expression. These results indicate that mTOR inhibition during M2 differentiation did not completely change polarization of these cells to M1 but rather induced an M1-like cell type as we could show that mTOR inhibited M2 did not develop the same pro-inflammatory profile as M1 polarized MPh.

In mature bovine moDC, mTOR inhibition increased expression of co-stimulatory CD80, as previously described in other species^13^ and in a previous experiment using bovine moDC generated from pregnant, non-lactating cows^45^. In an in-vitro model comparing bovine moDC from cows in different stages of gestation and lactation, Pomeroy et al.^9^ showed an increase of CD80 and MHCII in moDC from cows 4–6 days post-calving compared with moDC from mid and late gestation cows. Our results indicate that mTOR inhibition could be a mechanism contributing to this postpartum change in expression of co-stimulatory molecules. Complete mTOR pathway inhibition with Torin-1 in our model also increased expression of pro-inflammatory cytokines (*IL12A, IL12B, IL1β, IL6*) in *E. coli*- matured moDC which is consistent with findings in other species^19,46^.

As hypothesized, our study found effects of mTOR inhibition on immune phenotype, function, and cytokine profile of the cell types under study, albeit results differed between incomplete (RAPA) and complete (Torin-1) mTOR-pathway inhibition. Rapamycin, a naturally occurring first-generation mTOR inhibitor, interacts specifically with mTORC1, thereby inhibiting some of its functions^47^. However, rapamycin inhibition of mTORC1 shows feedback regulation, whereby mTORC1 4E-BP1 substrate phosphorylation is partially recovered over time^47,48^. Torin-1 is a second-generation mTOR inhibitor that reduces the kinase activity of both mTORC1 and mTORC2, blocking mTORC1 more effectively^47^ by evading the feedback loop and rapamycin-resistant functions of mTORC1 that complicates the use of rapamycin^49^. In general, the two inhibitors behaved similarly in our model with more pronounced changes in Torin-1-treated cells, which is encouraging to enhance our understanding of mTOR inhibition in bovine immune cells. Some effects, however, differed between RAPA and Torin-1. Most remarkably in this context we found opposing effects of the two inhibitors on expression of *IL12A* in M1 and M2 MPh, where RAPA induced up-regulation but treatment with Torin-1 lead to down-regulation*.* Additionally, we observed opposing effects of RAPA and Torin-1 on MHCII expression in naïve and mature moDC with RAPA increasing expression in naïve moDC, and Torin-1 decreasing MHCII expression in mature moDC. In a previous study, both rapamycin as well as PP242, a pan-mTOR inhibitor similar in action to Torin-1, increased MHCII expression, albeit not statistically significant for PP242^45^. Complete pathway inhibition by mTORC1/2 inhibitors such as Torin-1 may elicit different effects on moDC in our model than possibly incomplete mTORC1-specific inhibition by rapamycin as previously discussed by Weichhart et al.^12^. While mTORC1 regulates a cell´s growth and autophagy in response to nutrients, mTORC2 mediates other cell functions such as cytoskeletal organization^50^. Alternatively, effects of the type of mTOR inhibition might be cell-type specific, or inhibitor effects might be dose-dependent, and this was not investigated in our current work. In a recent study on mTOR-driven aging, authors showed that at low concentrations mTORC1/2 inhibitors acted like rapamycin, and that was particularly true for Torin-1 and PP242, although effects varied when concentrations increased^51^. A cytostatic effect in the model of Leontieva et al.^51^ was found at 30 nM for Torin-1 and 285 nM for PP242. Therefore, differences due to cell-type or dose-dependent responses to each type of inhibitor merit further attention in follow-up studies.

Ideally, the effects of mTOR inhibition could be tested in vivo in dairy cattle to help clarify the whole-body response to reduced mTOR activation. The use of mTOR inhibitors in this food-producing animal species would require authorization and removal of the animals and their products from the food chain. Alternatively, the mTOR pathway could be stimulated by supplying nutrients that are sensed by this pathway during times of deficit, either parenterally or through feed supplementation. Lastly, given that the greatest nutrient needs in the postpartum period are due to the requirements of lactation, cows that produce very little or no milk after calving could be compared with high producing cows regarding their mTOR activity. All aforementioned model systems carry their own limitations but could each contribute significantly to the further understanding of the mTOR pathway on postpartum immune phenotype of the dairy cow.

By using IFNG and LPS for polarization and stimulation of M1, and LPS and irradiated *E. coli* for maturation of moDC, we biased M1 and moDC towards a pro-inflammatory response^52,53^. However, gram-negative pathogens, specifically *E. coli*, are the most common infectious cause of severe inflammation in postpartum cows^7,54^.

We recognize our bovine model favored a Th1-promoting response a priori by using LPS for moDC maturation^52^. However, under such conditions we found further Th1-enhancing effects in mTOR-suppressed moDC compared with untreated controls. Decreased IL10 and increased IFNG and TNF concentrations in co-culture supernatants of Torin-1-treated moDC and MLR supports our hypothesis that inhibition of the mTOR-pathway in bovine moDC favors Th1 skewing. These findings are in line with our previous experiment showing increased TNF and decreased IL10 concentrations in rapamycin inhibited moDC, suggesting that mTOR inhibited moDC would favor a Th1 immune response^45^. When using mTOR-inhibited mature DC as stimulators for murine lymphocytes in co-culture, proliferating CD4^+^ T-cell populations displayed a Th1 phenotype^26,55^. We interpret observations in mTOR inhibited moDC in this model such that excessive nutrient deficit induced mTOR suppression in postpartum dairy cows might lead to a more pronounced Th1-dominated inflammatory response with stronger co-stimulatory potential of moDC and increased expression of pro-inflammatory cytokines.

**Figure 7:**
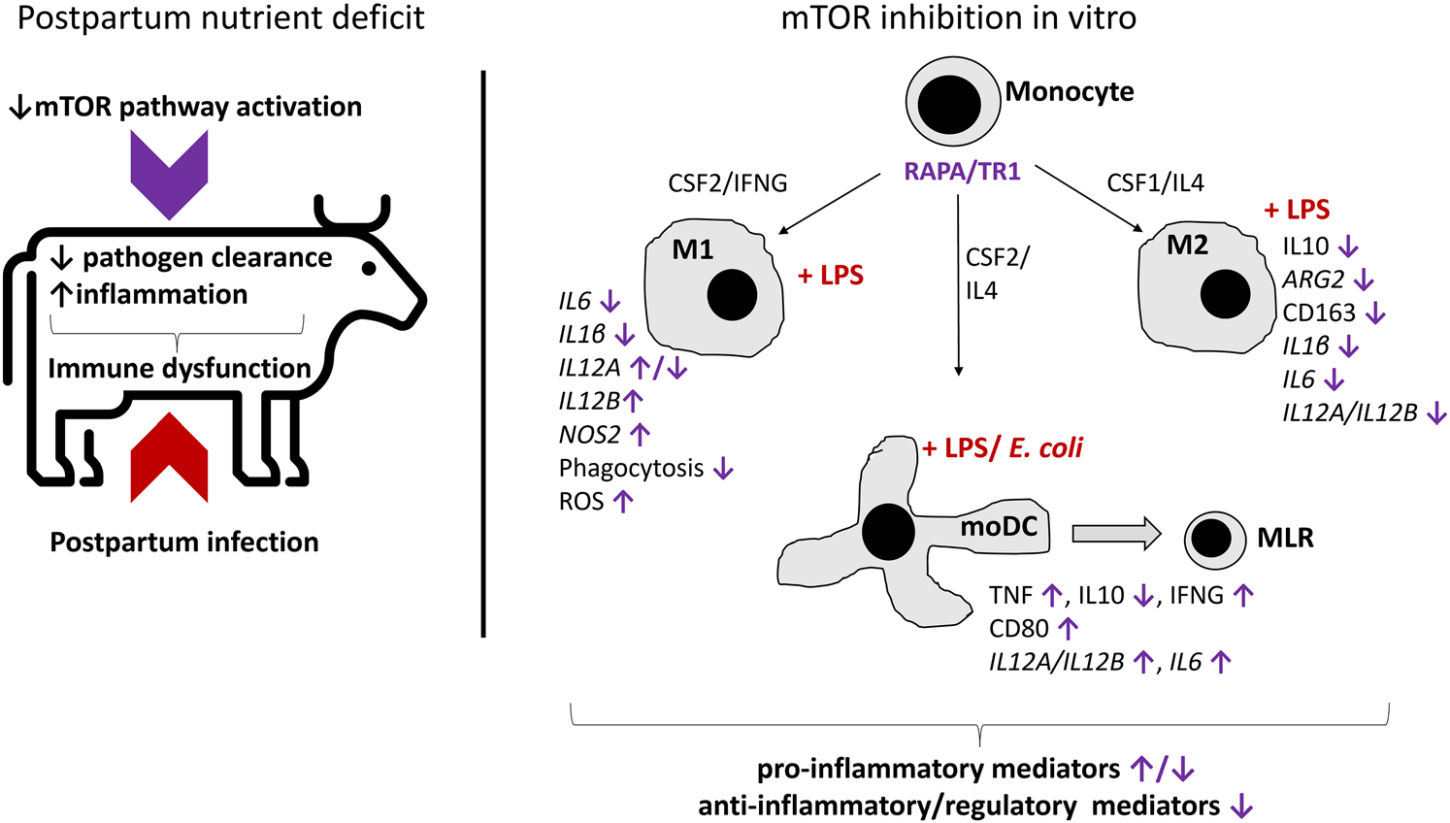
Summary schematic of hypothesized effects of mTOR inhibition during differentiation of myeloid cell populations in the presence of gram-negative bacterial components (*E. coli* lipopolysaccharide, LPS). Cows experience a nutrient deficit postpartum at the same time as immune dysfunction. We previously showed that the activation of the mTOR pathway is reduced in postpartum dairy cows. Summarized results are derived from *in-vitro* pharmacological mTOR inhibition with rapamycin (RAPA) or Torin-1 (TR1) in monocyte derived macrophages (M1, M2), dendritic cells (moDC), and co-culture of moDC with autologous mixed lymphocytes (MLR). We propose that the inflammatory response during nutrient deficit associated mTOR-pathway inhibition may result in the following effects: (1) increased production of reactive oxygen species (ROS) and expression of nitric oxide synthase 2 (*NOS2*) in M1, (2) decreased pathogen clearance by phagocytosis in M1, (3) selective regulation of pro-inflammatory mediators in M1 (*IL12A, IL12B, IL6, IL1β*), (4) increased expression of pro-inflammatory, Th1-promoting cytokines in moDC (*IL12A, IL12B, IL6,* tumor necrosis factor alpha, TNF) and a more potent activation of T cells by increased expression of co-stimulatory molecules (CD80) and cytokine profile changes (decreased IL10, increased TNF and IFNG) in moDC-MLR co-cultures, and (5) decrease in the M2 restorative phenotype hallmark features such as expression of arginase 2 (*ARG2*), surface CD163, and production of anti-inflammatory IL10. Overall, the observed changes indicate an inflammatory response that is heightened in magnitude and potentially in duration.

Summarizing the results of our study, we showed in this in-vitro model that reduced activation of the mTOR pathway alters the phenotype and cytokine profile in bovine monocyte-derived innate immune cells. An overview of these changes is given in Fig. 7. Selective reduction of cytokine expression and increased ROS production in LPS-stimulated M1, inhibition of M2 differentiation, and enhanced activation of moDC with a more pronounced Th1 response could contribute to a pro-inflammatory environment, delayed resolution of inflammatory response, and decreased pathogen clearance in vivo.

The resulting alteration of phenotype and cytokine profile in these key regulators of inflammation indicate that reduced activation of the mTOR pathway could off-set an adequate inflammatory response in postpartum dairy cows. A certain degree of increase in the reactive and pro-inflammatory immune response is considered physiological in the postpartum period^56,57^ and we previously showed that pro-inflammatory cytokines are upregulated postpartum in bovine whole blood leukocytes^17^. However, cows overexpressing pro-inflammatory cytokines over a prolonged period systemically and locally are at higher disease risk and show overall decreased efficiency of nutrient metabolism and milk production^7^. An excessive nutrient deficit might skew this balance towards heightened and prolonged inflammation with a decrease in pathogen clearance and restoration of homeostasis.

In conclusion, our results based on in vitro pharmacological mTOR inhibition indicate that suppression of mTOR-pathway activation caused by the lack of nutrient availability in postpartum dairy cows might contribute to the immune dysfunction observed during the critical postpartum time of dairy cows.

## Supplementary Information

Supplementary Information.

## Data Availability

The data that support the findings of this study are available, on reasonable request, from the corresponding author.
