# Supplementary material for: Inhibition of mTOR in bovine monocyte derived macrophages and dendritic cells provides a potential mechanism for postpartum immune dysfunction in dairy cows
Source: Sci Rep. 2022 Sep 5;12:15084. doi: 10.1038/s41598-022-19295-1 (PMC9445052; doi:10.1038/s41598-022-19295-1)
Supplement: Supplementary file 1 — Supplementary Information. [file 41598_2022_19295_MOESM1_ESM.pdf]

Supplemental File for manuscript:

Inhibition of mTOR in bovine monocyte derived macrophages and dendritic cells provides a potential mechanism for postpartum immune dysfunction in dairy cows

Anja S. Sipka[1,4*], Tawny L. Chandler[1,4], Thomas Weichhart[2], Hans-Joachim Schuberth[3], Sabine Mann[1*]

Anja S. Sipka and Tawny L. Chandler contributed equally to this work.

[1]Department of Population Medicine and Diagnostic Sciences, College of Veterinary Medicine, Cornell University, 231 Farrier Road, Ithaca, NY 14853, USA

[2]Center for Pathobiochemistry and Genetics, Medical University of Vienna, Währinger Straße 10, 1090 Vienna, Austria

[3]Institute for Immunology, University of Veterinary Medicine, Buenteweg 2, 30559 Hannover, Germany

[4]These authors contributed equally.

[*]Corresponding authors: ass233@cornell.edu, sm682@cornell.edu

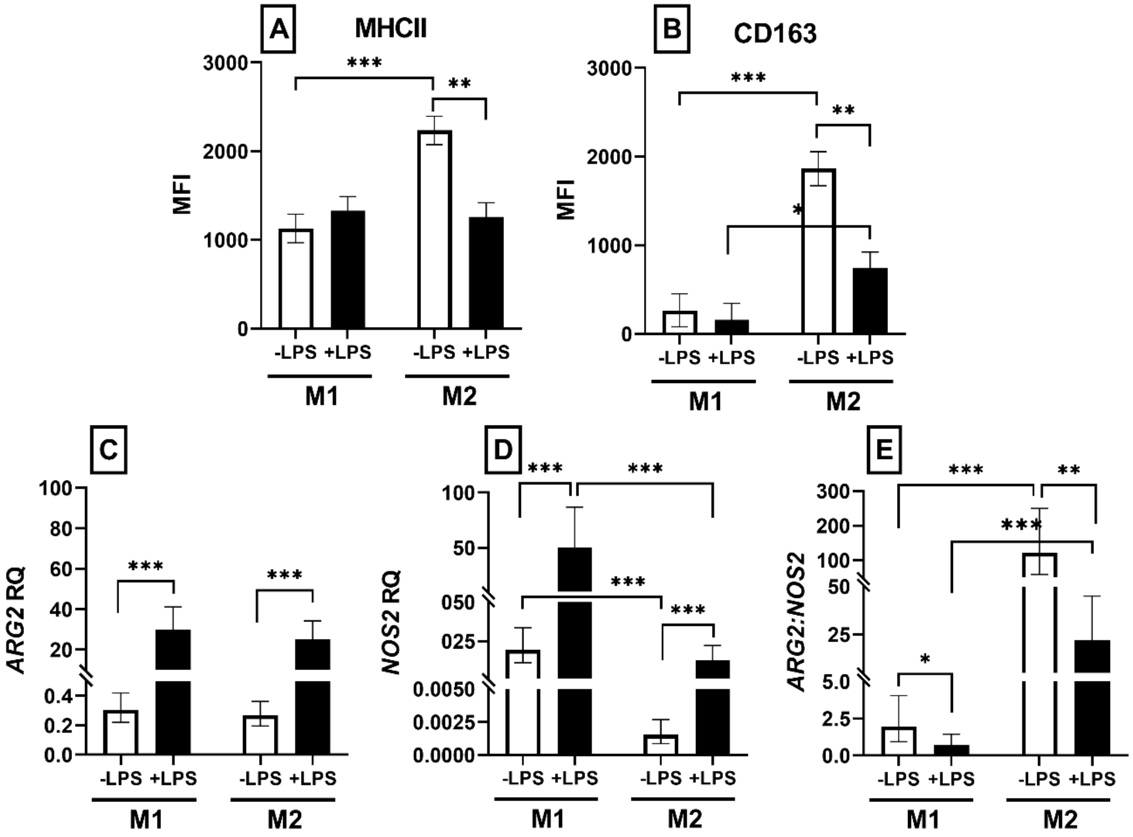

Supplemental Figure 1. Results of in vitro macrophage polarization from cell isolation of 12 cows. Cell surface staining of MHCII (**A**) and CD163 (**B**) following $CD14^+$ monocyte polarization with 10 ng/mL IFNG and 20 ng/mL CSF2 for 3 days to drive M1 macrophage polarization (M1), or 20 ng/mL IL4 and 20 ng/mL CSF1 for 3 days to drive M2 macrophage polarization (M2), before incubation for 16 h with vehicle (-LPS) or stimulated with LPS at 100 ng/mL (+LPS). Abundance of mRNA of *arginase 2* (*ARG2*, **C**) and *inducible nitric oxide synthase* (*NOS2*, **D**), and their ratio (**E**) in polarized cells that were stimulated with LPS for 4 h. Data for surface maker expression are presented as LS means ± SE, and data for mRNA abundance and ratio are presented as geometric mean and backtransformed 95% confidence interval. Pairwise comparisons between macrophage type and stimulus are shown with Bonferroni-adjustment for multiple comparisons: $P \leq 0.10$ ($^{\#}$), $P \leq 0.05$ (*), $P \leq 0.01$ (**), $P \leq 0.0001$ (***).

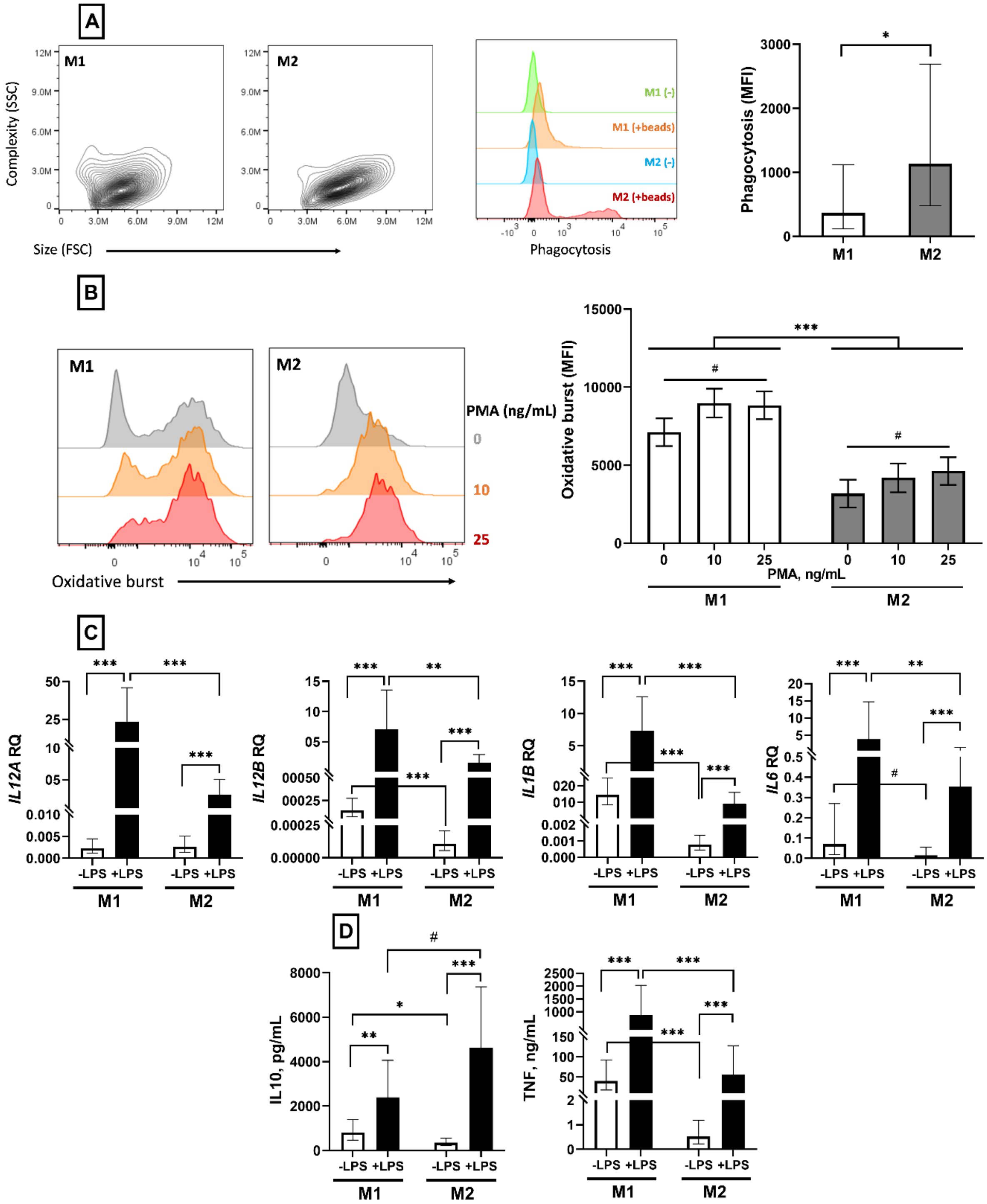

Supplemental Figure 2.

Supplemental Figure 2. Mean fluorescence intensity (MFI) of polarized macrophages following $CD14^+$ monocyte polarization with 10 ng/mL IFNG and 20 ng/mL CSF2 for 3 days to drive M1 macrophage polarization (M1), or 20 ng/mL IL4 and 20 ng/mL CSF1 for 3 days to drive M2 macrophage polarization (M2) before 1 h incubation with pHrodo bioparticles to measure cellular phagocytosis. Contour plots show size and complexity in forward (FSC) and side scatter (SSC) of M1 and M2. Histograms show fluorescence of pHrodo beads in M1 and M2 (**A**). To measure oxidative burst M1 and M2 were incubated with 10 µM H_2DCFDA fluorescein loading dye for 15 min and stimulated with phorbol myristate acetate (PMA) at 0, 10, or 25 ng/mL for 15 min. Histograms show fluorescence of H_2DCFDA for M1 and M2 stimulated with PMA (10, 25 ng/mL) or left as unstimulated control (**B**). Phagocytosis data are presented as geometric mean and backtransformed 95% confidence interval. Oxidative burst data are presented as LS means ± SE. Abundance of mRNA of *IL12A*, *IL12B*, *IL1B*, and *IL6* (**C**) in polarized macrophages following incubation with vehicle (-LPS) or stimulation with 100 ng/mL LPS for 4 h (+LPS). Concentration of cytokines IL10 and TNF (**D**) in cell culture supernatant for polarized macrophages that were stimulated with LPS for 16 h. Data from cell isolation of 12 cows are presented as the geometric mean and backtransformed 95% confidence interval. Pairwise comparisons between macrophage type and stimulus are shown with Bonferroni-adjustment for multiple comparisons: $P \leq 0.10$ ($^{\#}$), $P \leq 0.05$ (*), $P \leq 0.01$ (**), $P \leq 0.0001$ (***).

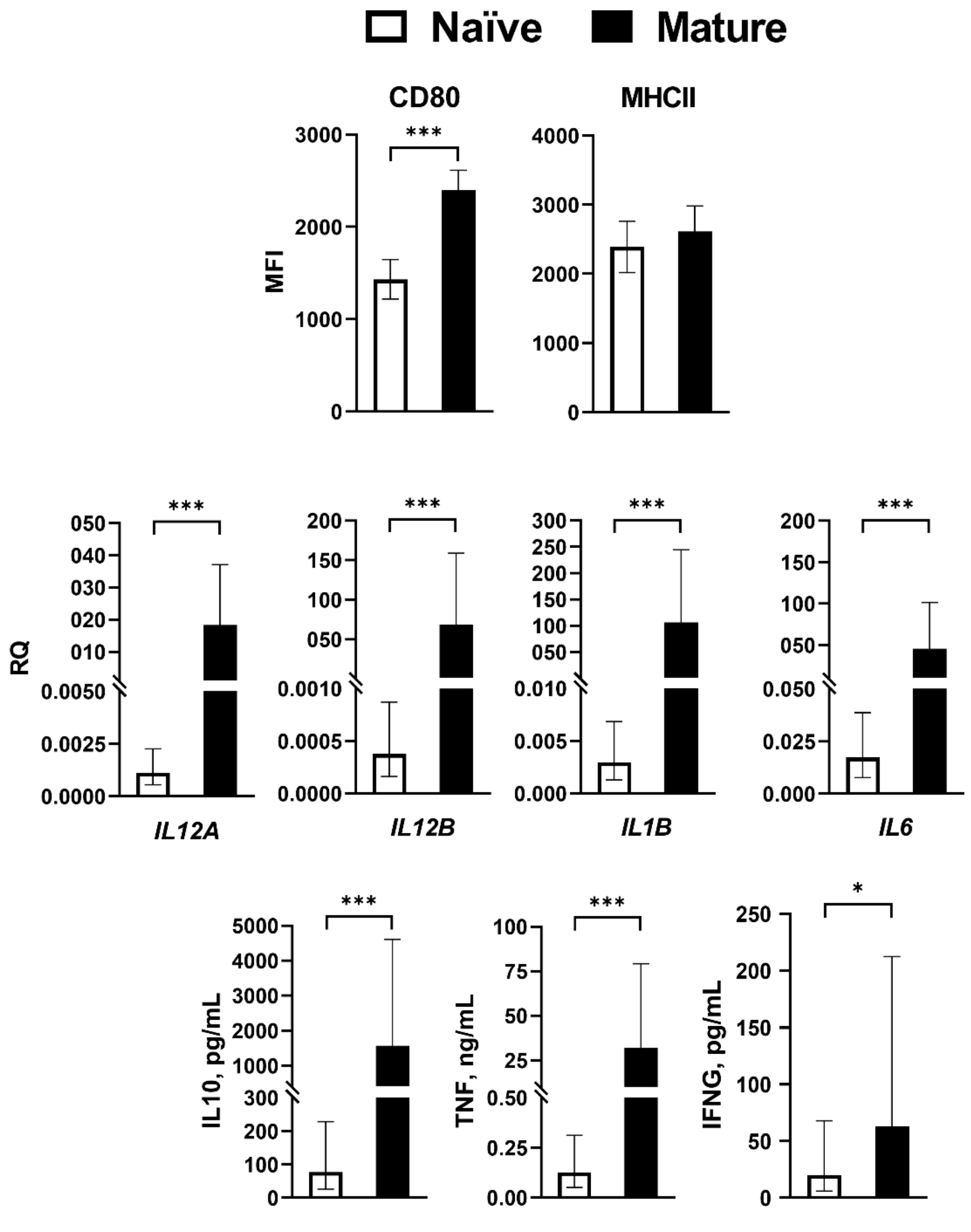

Supplemental Figure 3. Results of monocyte-derived dendritic cell (moDC) differentiation protocol from cell isolation of 12 cows. Cell phenotype and cytokine responses following $CD14^+$ monocyte differentiation with 20 ng/mL CSF2 and IL4 for 7 days to generate naïve moDC (Naïve) before cells were matured with 100 ng/mL LPS (Mature) for 16 h to measure surface marker expression and concentration of IL10, TNF, and IFNG in cell culture supernatant, or matured for 4 h to quantify cellular mRNA abundance of *IL12A*, *IL12B*, *IL1B*, and *IL6*. Data for surface maker expression are presented as LS means ± SE, and data for mRNA abundance and cytokine production are presented as geometric mean and backtransformed 95% confidence interval. Difference between naïve and mature moDC were tested by ANOVA: $P \leq 0.10$ ($^{\#}$), $P \leq 0.05$ (*), $P \leq 0.01$ (**), $P \leq 0.0001$ (***).
